# The Evaluation of Anemia Among Stunted Children Aged 6–24 Months in Bandung District, West Java, Indonesia

**DOI:** 10.3390/children12050638

**Published:** 2025-05-15

**Authors:** Susi Susanah, David Marcelius, Lulu Eva Rakhmilla, Rini Rossanti, Sindy Amalia Febrianti, Siti Sakinah, Winyarti Winyarti, Safira Satyani Lutfia, Raden Tina Dewi Judistiani, Dida Akhmad Gurnida, Budi Setiabudiawan

**Affiliations:** 1 Department of Child Health, Hematology-Oncology Division, Dr. Hasan Sadikin General Hospital/Faculty of Medicine, Universitas Padjadjaran, Bandung 40161, Indonesia; sindy.amalia190291@gmail.com; 2 Department of Child Health, Faculty of Medicine, Universitas Padjadjaran, Bandung 40161, Indonesia; david_marcel08@yahoo.com (D.M.); siti16076@mail.unpad.ac.id (S.S.); winyarti23001@mail.unpad.ac.id (W.W.); safira16007@mail.unpad.ac.id (S.S.L.); 3 Department of Public Health, Epidemiology and Biostatistic Division, Faculty of Medicine, Universitas Padjadjaran, Bandung 40161, Indonesia; lulu.eva.rakhmilla@unpad.ac.id; 4 Department of Child Health, Nephrology Division, Dr. Hasan Sadikin General Hospital/Faculty of Medicine, Universitas Padjadjaran, Bandung 40161, Indonesia; rini.rossanti@unpad.ac.id; 5 Department of Obstetric and Gynecology, Dr. Hasan Sadikin General Hospital/Faculty of Medicine, Universitas Padjadjaran, Bandung 40161, Indonesia; tina.d.judistiani@unpad.ac.id; 6 Department of Child Health, Nutrition and Metabolic Disease Division, Dr. Hasan Sadikin General Hospital/Faculty of Medicine, Universitas Padjadjaran, Bandung 40161, Indonesia; dida.gurnida@unpad.ac.id; 7 Department of Child Health, Allergy-Immunology Division, Dr. Hasan Sadikin General Hospital/Faculty of Medicine, Universitas Padjadjaran, Bandung 40161, Indonesia; budi.setiabudiawan@unpad.ac.id

**Keywords:** anemia, co-occurrence, iron deficiency, risk factors, stunting

## Abstract

**Background/Objectives**: Anemia, particularly iron-deficiency anemia (IDA), and stunting remain notable early childhood public health challenges in Indonesia; however, studies are still scarce. This study aimed to determine the co-occurrence of anemia and stunting (CAS), their prevalence, and the associated factors, as well as to describe the erythrocyte parameters. **Methods**: Approximately 2200 children aged 6–24 months were identified by midwives to have problematic nutritional status at Bandung Regency, West Java, Indonesia. These children were included in the population frame for a cohort study of vitamin D deficiency, vitamin D binding protein, and its impact on neurodevelopmental functions. A cross-sectional study was nested in the cohort study. The subjects were selected by stratified random sampling of 270 villages to meet the required number of samples. Medical doctors reassessed the anthropometric measurements and performed guided interviews to collect associated factors for IDA and CAS. Erythrocyte profiles of the children were examined from venous blood. **Results**: One hundred and ninety-four subjects were included in the analysis, among which 54.1% were stunted. Anemia was present in 40.7% of the subjects, largely due to IDA (87.3%). A wasting child and the factor of low paternal education (up to elementary school) were associated with IDA (aOR of 7.12 and aOR of 3.32, *p* < 0.05, respectively). Co-occurrence of anemia and stunting was found in 41/194 (21.1%) subjects, but it did not show significant association. **Conclusions**: Anemia and stunting were prevalent among children aged 6–24 months, but no associations were found between anemia and stunting in this study. Iron deficiency was the main cause of anemia, and it was associated with wasting and low paternal education.

## 1. Introduction

Anemia, as a major indicator of overall health, remains a significant public health issue, particularly in children, due to its association with significant morbidity related to growth and development disorders, and even mortality [1]. Globally, approximately 25% of the population experiences anemia, and 42.6% of those are children [2]. In Indonesia, anemia affected 23.7% of the population and 38.5% of children under 5 years old in 2018 [3]. In 2020, a study of Indonesian elementary students with a smaller sample found similar findings, with 27% being anemic [4]. Various etiologies and risk factors have been identified; however, the most common cause was nutritional anemia, particularly iron-deficiency (ID), which was responsible for approximately 10–60% of anemia cases [1]. Anemia frequently coexists with malnutrition, and the presence of both conditions during early life leads to considerable long-term health, development, and social consequences later in life [4,5].

Stunting, as a consequence of chronic malnutrition, is also one of the persistent problems faced by children in developing countries, including Indonesia [6]. According to data from the Indonesia Basic Health Research conducted in 2018, stunting and severe stunting were prevalent in 30.8% of toddlers and 29.9% of infants under 2 years old [3]. The majority of stunting cases occur in the first 1000 days or before the age of 2 years, and in this period, stunting predicts poorer cognitive and educational outcomes in later childhood and adolescence [7].

The co-occurrence of anemia and stunting (CAS) is highly possible and has been reported in several studies [8,9,10,11,12]. The proposed mechanism that could explain this coexistence is that stunting could cause anemia by decreasing the erythropoietin production [13]. A study found that children with stunting have a 2.3 times higher risk of anemia than those without stunting [14]. Despite the interrelationship, several studies have conflicting results, stating that anemia and stunting are two independent conditions [15,16,17,18,19,20]. Given these inconsistent research findings, further study is needed to strengthen the interrelationship.

Anemia and stunting in children, whether they occur alone, together, or in combination with other conditions, must be promptly diagnosed to initiate appropriate treatment. In anemia cases accompanied by stunting, treating anemia is expected to improve the stunting condition. Enhancing both nutrition and anemia in children is essential to ensure a better quality of life and produce a high-quality generation. To the best of our knowledge, studies on CAS are still limited in Indonesia. Therefore, this study aimed to determine the prevalence of anemia, stunting, and their co-occurrence among children aged 6–24 months, along with the associated sociodemographic data, as well as to explore the etiology of anemia using erythrocyte parameters, including Ret-He.

## 2. Materials and Methods

### 2.1. Study Design, Setting, and Participants

In 2022, the Bandung Regency Health Office, West Java, Indonesia, carried out a routine monitoring program for child growth for all 270 villages in the regency. Approximately 2200 children aged 6–24 months were identified by midwives to have problematic nutritional status. These children were included in the population frame for an Academic Leadership Grant (ALG) research, a cohort study of vitamin D deficiency, vitamin D binding protein, and its impact on neurodevelopmental functions.

**Figure 1:**
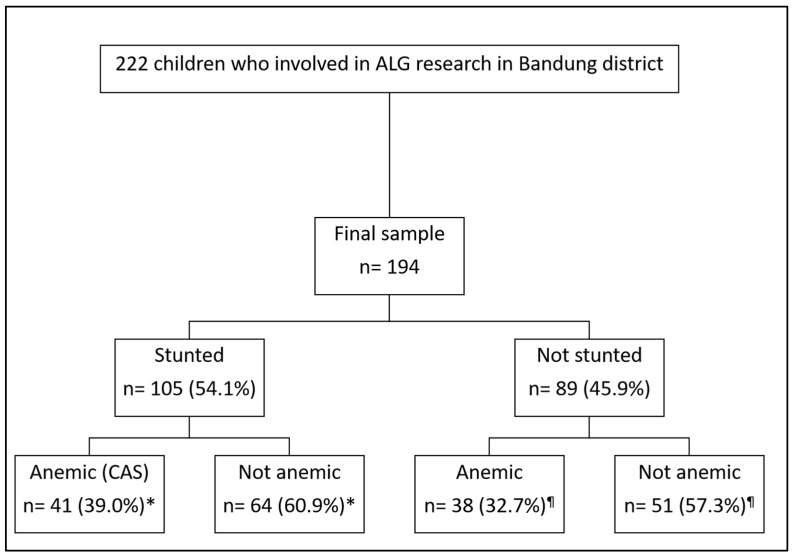
Sample selection. Note: * proportion among the participants with stunting; ¶ proportion among the participants without stunting.

A cross-sectional study was nested in the cohort study. A sample of 222 children was selected by stratified random sampling from the population frame. Exclusions were applied to children who had congenital defects or chronic diseases (tuberculosis, malignancy, and hepatitis) during the second screening, which was performed by medical doctors as field researchers. Further exclusion occurred due to unavailability of laboratory examination results. A total of 194 children were included in the final dataset (Figure 1). The selected paired children and their mothers as the subjects of this study were invited to participate. Information regarding the study was explained to the mothers, and written consent forms were obtained.

### 2.2. Data Collection

The second-stage screening and examinations took place at the “posyandu”, the community-based health posts in each village where monthly health checks and free immunizations for children take place. The nearest posyandu to the address of the paired subjects was selected. On a designated date, a team of medical doctors and an expert nurse were present at the posyandu.

The medical doctors, as field researchers, were trained in standardized methods for physical examinations and guided interviews for this study. The nutritional status was assessed by anthropometric measurements. The procedure and result interpretation followed the guidelines for child growth from the World Health Organization (WHO), 2006 [21]. Body weight measurements for children under 2 years old were carried out using Elitech DIGIT-ONE BABYÒ/TD 05219B3340 (accredited by the Ministry of Health of the Republic of Indonesia, No. 10901410291). The interview used a guided questionnaire for collecting data on previous history at birth, breastfeeding, and illness. No questionnaire validation was applied.

The nurse took venous blood samples from each selected child. The blood was kept in standardized cooled containers for transport to the Hasan Sadikin Hospital laboratory. It took one to two hours of transport time before these samples reached the laboratory facility. Analysis of 36 hematological parameters was conducted using a Sysmex XN-1000 hematology analyzer to determine the hemoglobin (Hb) level, red blood cell (RBC) counts, Ret-He, and erythrocyte indices (mean corpuscular volume (MCV), mean corpuscular hemoglobin (MCH), mean corpuscular hemoglobin concentration (MCHC)).

### 2.3. Diagnostic Criteria

Anemia was defined as an Hb level <11 g/dL. Iron-deficiency anemia (IDA) was characterized by the presence of microcytic hypochromic anemia (MCV < 80 fL, MCH < 27 pg, MCHC < 31 g/dL) and Ret-He concentration according to age (for children aged 6–24 months, the cut point is <27.65) as recommended by the American Academy of Pediatrics (AAP) [22,23,24]. Initial assessment to differentiate from other etiologies of microcytic hypochromic anemia, such as thalassemia minor/trait, followed the anemia/thalassemia screening guidelines by the Indonesian Ministry of Health, which were based on the Mentzer index (MCV/RBC counts) >13 and the Shine and Lal index (SLI). A value of SLI > 1530 may suggest IDA, which is calculated by the formula MCV × MCV × MCH/100. A child was diagnosed with CAS, as the main variable of interest, if anemia and stunting were present.

Stunting was defined as the growth curves for body length <− 2 standard deviations or body height <− 2 standard deviations according to sex and age, according to the 2006 WHO growth standards [25]. A child was diagnosed as (1) wasted if weight-for-length/height was ≤−2 SDs/≥−3 SDs of the median or (2) severely wasted if weight-for-length/height was <−3 SDs of the median.

During the interview, the following data were requested: birth weight, prematurity (preterm or term), and duration of breastfeeding only. Data were then recoded further. Birth weight was categorized as normal if it was ≥2500 g and low if it was <2500 g. Prematurity was recoded as preterm, and exclusive breastfeeding was met if the baby was breastfed only for at least 6 months. The age of children was based on complete months, while the parents’ age was based on complete years. Classification of parental education was based on whether or not they finished each level. The household income was calculated as combined parental income and recoded based on the Bandung Regency’s regional minimum wage standard cutoff for categorical analysis, which was Rp 3,241,000 (USD 216) in 2021, regardless of the number of family members. Information regarding children’s daily consumption was obtained by dietitians through dietary recall.

Potential predictors associated with CAS were identified based on the literature from the UNICEF’s conceptual framework of the determinants of child undernutrition and the WHO’s conceptual framework of anemia etiology.

### 2.4. Statistical Analysis

The Shapiro–Wilk test was used to check for normality in continuous data. Descriptive statistics were presented as the means ± standard deviations for normally distributed continuous data, or as the medians (interquartile range (IQR)) for non-normally distributed continuous data. Categorical data were presented as frequencies and percentages, and for the evaluation between the categorical groups, the chi-squared test was used to obtain the significance of the difference. When the criteria for the chi-squared test were not met, the Kolmogorov–Smirnov test was performed. The Mann–Whitney test was used to analyze numerical independent variables.

Stunting was defined as the primary dependent variable, and the differences between stunting risk factors are presented in Table 1. Iron-deficiency anemia (IDA) and its associated factors are presented in Table 2. Co-occurrence of anemia and stunting (CAS) was also analyzed as a dependent variable, as presented in Table 3, and the multivariate logistic regression analysis of the associated factors is presented in Table 4. The results of the multiple logistic regression are expressed as odds ratios (OR) and 95% confidence intervals (95% CI), with adjustments to control potential confounders. The statistical analysis was performed using STATA/BE 17.0.

### 2.5. Ethics

Ethical approval was obtained from the Ethics Committee of the Medical Faculty of Universitas Padjadjaran/Dr. Hasan Sadikin General Hospital, Bandung, reference number LB.02.01/X.6.5/1./2022. Written informed consent was obtained from all parents of the patients voluntarily, and this study was conducted according to the principles of the Declaration of Helsinki.

## 3. Results

### 3.1. Sociodemographic Characteristics of the Study

Of the 194 children included in this study, 54.1% had stunting. Overall, there were more boys than girls (111 [57.2%] vs. 83 [42.8%]), and the mean age of the participants was 16.7 ± 4.7 months. Other demographic characteristics of the participants with and without stunting are presented in Table 1. The stunting group recorded a higher mean age, showing a significant difference between the stunting and non-stunting groups (*p* = 0.004). Bivariate analysis also revealed an association between stunting and birth weight (*p* = 0.027) and weight-for-age (*p* < 0.001). However, no significant differences or associations were noted between dietary and socioeconomic factors and stunting (*p* > 0.05).

### 3.2. Prevalence of Anemia and CAS

The prevalence of anemia was 40.7% (*n* = 79), and 69 out of 79 (87.3%) were diagnosed with IDA. The mean Hb level was 11.2 ± 1.9 g/dL; mean hematocrit, 36.1% ± 3.7%; mean MCV, 73.2 ± 7.3 fL; mean MCH, 22.7 ± 3.1 pg; and mean MCHC, 31.0 ± 1.9 g/dL. The observed prevalence of CAS among the participants was 41/194 (21.1%). The prevalence of IDA was high in the CAS and non-CAS groups.

### 3.3. Factors Associated with IDA

Multivariate logistic regression showed that stunting, wasting, and paternal education were associated with IDA (Table 2). The wasting group was seven times more likely to develop IDA (OR, 7.12 (1.60–31.71)) than children with normal nutritional status. Moreover, the odds of IDA were three times higher in children whose fathers had only primary education (OR, 3.32 (1.35–8.20) than in those who had high school education. In contrast, this study found that stunted children were less likely to develop IDA (OR (95% CI), 0.42 (0.19–0.94)).

### 3.4. Factors Associated with the Co-Occurrence of Anemia and Stunting (CAS)

Table 3 shows that the associated factors for CAS were the mean hemoglobin level, IDA, and weight-for-age.

Further multivariate logistic regression analysis of the factors that have a significant association with CAS are presented in Table 4.

Based on multivariate logistic regression (Table 4), there were no predictor factors associated with CAS in this study (*p* > 0.05).

## 4. Discussion

Despite a decline in prevalence over the last several decades, stunting still poses a major issue faced by children under 5 years of age [18]. Over half (52%) of the stunted children under 5 years old in 2022 are from Asia, whereas over a third (43%) are from Africa [19]. The largest percentage (53.7%) of Asia’s 76.6 million stunted children under 5 are from South Asia [19]. The WHO reports that Indonesia ranks third among the countries of Southeast Asia/South-East Asia Region (SEAR) with the largest population of children with stunting [7]. In the present study, the prevalence of stunting was high (54.1%). This was higher than the reported rates of 21.6% and 20.2% in the Indonesia Nutritional Status Survey in 2022 and West Java, respectively [20]. The prevalence was also higher than the global estimate of stunting in children under the age of 5 as reported in UNICEF’s, the WHO’s, and the World Bank’s joint estimates in 2022 (22.3%) [26].

In 2020, UNICEF published a revised framework on the determinants of maternal and child nutrition to facilitate targeted intervention and strategy for 2020–2030 [22]. The present study used this framework to determine the potential factors associated with stunting and nutritional anemia. Bivariate analysis found that age, birth weight, and weight-for-age were significantly associated with stunting. Based on a previous study, the prevalence of stunting tends to increase until around 28 months and starts to decrease between the ages of 30 and 59 months [23]. The stunting rate is higher in children with LBW (52.64%) [24,25]. Another study by Sartika et al. conducted in Sambas District, West Kalimantan Province of Indonesia, reported that children with LBW were four times more likely to be stunted [25]. Low birth weight often indicates prenatal nutrition insufficiency and maternal health during pregnancy [25,27]. A child’s weight-for-age also showed a significant association with stunting, where an underweight child was 16.74 times more likely to be stunted [28]. Underweight is one of the malnutrition indicators that could depict the nutritional status of a child; hence, the long-term effect could lead to delayed growth, including linear growth. Moreover, Beal et al. found that nonexclusive breastfeeding, low household socioeconomic status, premature birth, short birth length, and low maternal height and education were important determinants of child stunting in Indonesia [29].

In this study, the prevalence of anemia (41.7%) fell into the category of a severe public health problem [1]. This was higher than the results of the Basic Health Research, which reported a 38.5% prevalence of anemia among children under age 5 in 2018 [3]. The majority of anemia cases occur in low- to middle-income countries; these populations are more likely to live in rural areas and lower-class homes and not have had a formal education [18]. Anemia is thought to afflict 40% of all infants aged 6–59 months, 37% of pregnant women, and 30% of women aged 15–49 years worldwide [18]. In the present study, 87.3% of the participants had IDA, and factors associated with IDA were nutritional status, particularly height-for-age and weight-for-length, and father’s education. Iron is crucial in all tissues in a child’s developing body [30]. Because of impaired iron function, ID with or without anemia in the first 1000 days of life disrupts immune function and brain development, which leads to growth and development delays [31]. Although anemia can be managed, the neurocognitive deficits caused by ID could remain permanent [31]. Diagnosing ID is crucial in preventing long-term complications; hence, the etiology of anemia should be established as soon as possible.

Conventionally, the diagnosis of IDA was based on the presence of microcytic hypochromic anemia and abnormal iron status, such as transferrin saturation <10% and serum ferritin level <12 ng/mL [32]. Nevertheless, these measurements are not practical for the community setting because of facility constraints (require several blood samples, high cost) and biological variability such as diurnal variations, diet, and inflammation. Thus, another parameter that can overcome this problem is required. Many studies have reported the correlation of Ret-He with iron status in diagnosing IDA so that it could be used as a marker of ID, suggesting it to be cost-effective, simple, and practical. This parameter measures the Hb content in the reticulocytes that have just been released from the bone marrow. It is expected to represent the real iron content in the bone marrow so that it can be used to detect ID earlier. Currently, the AAP recommends Ret-He as a parameter to detect ID [33]. The AAP considers IDA in children as an Hb level of <11 g/dL and one of the two criteria: (i) serum ferritin and CRP measurement or (ii) Ret-He measurement. It is also recommended measuring either (i) serum ferritin and CRP or (ii) Ret-He to screen for ID [33]. Ret-He directly measures the recent functional availability of iron in erythrocytes [33]. Prior studies showed that Ret-He is an early and sensitive marker for the diagnosis of ID and is not affected by inflammation, diurnal variation, malignancy, or anemia of chronic disease [33]. Unlike other biochemical assays, Ret-He can be measured together with a complete peripheral blood test; therefore, it does not require additional blood tubes and is provided at no additional cost [34]. In Indonesia, no standardized agreement has been achieved on the reference values for the exact normal limit of Ret-He levels for each age group. Various studies have utilized cutoff points from previous research. Ideally, a diagnostic tool should have high sensitivity to exclude diseases and high specificity to include diseases. Based on previous studies, this study adopted 27.65 pg as the cutoff point of Ret-He, which has 91.7% sensitivity and 78.3% specificity [35].

Stunting could impair the synthesis of Hb and red blood cells through mechanisms such as reduced erythropoietin production brought on by proinflammatory cytokines and inadequate consumption of vital nutrients such as iron, vitamins, and minerals, which exacerbates anemia [13]. Leptin levels are also low in children with stunting, and wasting negatively affects erythropoiesis [36]. Despite the linkage of pathological processes, a previous study showed that stunting and anemia are two independent and unrelated events [15]. Previous studies highlighted the association between anemia and stunting, with stunting considered a risk factor for developing IDA [8,9,37]. However, there are inconsistent results across studies. Most studies were conducted with a cross-sectional design, which cannot produce evidence of causality. As with the findings in this study, stunted children were less likely to develop iron-deficiency anemia. This contradicts previous studies that reported that stunted children had lower Hb and were 1.4 times more likely to have IDA [38,39]. Gosdin et al., Castejon et al., and Albalak et al. reported similar results, concluding that anemia and stunting are independent of each other and are better addressed by tailored interventions [15,16,17]. Data regarding the advantages of iron supplementation for children who do not have a high risk of IDA are still insufficient and inconsistent.

Although anemia and stunting were not found to be related in this study, these conditions were expected to occur simultaneously to a greater degree than by chance [15]. Since anemia and stunting most commonly occur in the first 1000 days or before 2 years of age, they tend to coexist. This study revealed a CAS prevalence of 21.1%. Our findings were higher than those of several studies, such as 5.6% in Peru, 9.9% in Egypt, 5.7% among children aged 6–11 years in Indonesia, and 5.7% among adolescents aged 13–15 years old in Indonesia [13,40,41]. A higher prevalence of CAS was found among children of preschool age in Ethiopia, ranging from 17.8% to 24.4% [8,9,10,42,43,44]. A similar finding was reported in India with 21.5% [15]. In Myanmar, a study found that three out of every five children with stunting were anemic [12]. While stunting and anemia alone present a serious threat to children’s lives and the health system, CAS would be considerably more harmful [8]. Early identification of and intervention for CAS is crucial to prevent the potential double burden and long-term consequences in later life.

In 2018, the Indonesian Health Ministry published the multisectoral strategy for accelerating stunting reduction, including food fortification and supplementary food as part of the nutritional intervention [45,46]. Supplementary food was adjusted to local products, and it was managed by local authorities and health centers. Effectiveness and implementation rate of this program across Indonesia remain debatable, and further studies are necessary to conclude that food fortification is effective in tackling stunting and decreasing the risk of anemia, especially IDA. Furthermore, the Government of Indonesia has pledged to combat stunting and wasting, establishing challenging goals to be accomplished by 2024 and 2025 [26]. A significant amount of effort has already been made to help reach these goals, motivated by a strong political commitment. The Ministry of Health has commenced operations in order to increase access to care for nutrition, and efforts have been increased, with an emphasis on stopping stunting in every province [26].

One of the strengths of this study was the analysis of locally representative data, as the participants were chosen from a district consisting of several subdistricts and villages. However, the study limitations related to the data and the methodology must be addressed. This study employed a cross-sectional design, which precluded the inclusion of the lapse period between the predictors and the result variables. As a result, any conclusions about causality should be regarded as only associations. Furthermore, other etiologies of anemia were not elaborated specifically. Despite these limitations, this study offers vital evidence that can be used to encourage policy and public health practice initiatives to further analyze and consider better comprehensive programs regarding stunting and anemia, particularly in children.

## 5. Conclusions

A high prevalence of anemia, stunting, and CAS was observed among children aged 6–24 months. As predicted, ID was the main cause of anemia. Although the association between stunting and anemia was not statistically significant, both conditions are known to share overlapping risk factors and often occur simultaneously. Therefore, integrated intervention strategies are essential to effectively address both nutritional issues within the same target population. Prospective studies are recommended to further investigate the causal mechanisms linking stunting and anemia, including the possibility of a bidirectional relationship.

## Figures and Tables

**Table 1:** Characteristics of the subjects (*n* = 194).

Characteristics	Stunting (*n* = 105)	No Stunting (*n* = 89)	*p*
Subjects (*n* = 194)			
Age (months): mean ± SD	18 ± 7	15.7 ± 4.7	0.004 *
Sex			0.788
Male	61 (58.1)	50 (56.2)	
Female	44 (41.9)	39 (43.8)	
Birth weight			0.027 *
Normal	82 (78.1)	80 (89.9)	
Low birth weight	23 (21.9)	9 (10.1)	
Hemoglobin	11.2 ± 1.9	11.3 ± 1.6	0.606
<11 g/dL	41 (39.0)	38 (42.7)	
≥11 g/dL	64 (61.0)	51 (57.3)	
Iron-deficiency anemia			0.314
Yes	34 (32.4)	35 (39.3)	
No	71 (67.6)	54 (60.7)	
Meal frequency per day			0.050
3 times or more	61 (58.1)	51 (57.3)	
2 times	40 (38.1)	35 (39.3)	
Once	4 (3.8)	3 (3.4)	
Exclusive breastfeeding			0.430
Yes	74 (70.5)	58 (65.2)	
No	31 (29.5)	31 (34.8)	
Current formula milk consumption			0.826
Yes	31 (34.8)	35 (33.3)	
No	70 (66.7)	58 (65.2)	
Weekly meat consumption	2 ± 2	2.2 ± 2.1	0.452
Weekly consumption of legumes and nuts	2 ± 6	2.8 ± 2.8	0.607
Weekly egg consumption	4 ± 5	4.2 ± 2.6	0.326
Weekly milk consumption	0 ± 7	2.7 ± 3.7	0.855
Mother’s age (years): mean ± SD	29 ± 12	29.9 ± 6.4	0.372
Gestational age			0.489
Term	91 (86.7)	80 (89.9)	
Preterm	14 (13.3)	9 (10.1)	
Father’s age (years): mean ± SD	33 ± 12	33.4 ± 7.6	0.372
Mother’s education			0.179
No education	1 (1)	1 (1.1)	
Primary school	40 (38.1)	21 (23.6)	
Junior high school	44 (41.9)	39 (43.8)	
Senior high school	18 (17.1)	25 (28.1)	
University	2 (1.9)	3 (3.4)	
Father’s education			0.060
No education	0 (0.0)	1 (1.1)	
Primary school	45 (42.9)	23 (25.8)	
Junior high school	35 (33.3)	30 (33.7)	
Senior high school	23 (21.9)	33 (37.1)	
University	2 (1.9)	2 (2.3)	
Family income (per month)			0.351
≥regional minimum wage standard	18 (17.1)	20 (22.5)	
<regional minimum wage standard	87 (82.9)	69 (77.5)	
Weight-for-age			<0.001 *
Normal	53 (50.5)	79 (88.8)	
Underweight	22 (21.0)	9 (10.1)	
Severely underweight	30 (28.6)	1 (1.1)	
Weight-for-length			0.351
Normal	73 (69.5)	75 (84.3)	
Overweight	1 (1.0)	0 (0.0)	
Wasted	14 (13.3)	7 (7.9)	
Severely wasted	17 (16.2)	7 (7.9)	

* Statistically significant.

**Table 2:** Multivariate logistic regression analysis of the factors that were considerably associated with IDA.

Variables	OR (95%CI)	*p*
Stunting	0.42 (0.19 to 0.94)	0.04 *
Age	1.02 (0.94 to 1.09)	0.69
Normal birth weight	0.55 (0.19 to 1.57)	0.26
Weight-for-age		
Normal	0.83 (0.22 to 3.15)	0.79
Weight-for-length		
Normal	3.40 (0.85 to 13.61)	0.08
Wasted	7.12 (1.60 to 31.71)	0.01 *
Meal frequency per day		
3 times or more	1.36 (0.68 to 2.72)	0.38
No exclusive breastfeeding	0.45 (0.19 to 1.02)	0.06
Formula milk consumption	0.75 (0.33 to 1.70)	0.49
Preterm gestational age	1.14 (0.34 to 3.76)	0.83
Mother’s education		
Primary school	0.79 (0.33 to 1.86)	0.59
Senior high school	0.83 (0.33 to 2.08)	0.69
Father’s education		
Primary school	3.32 (1.35 to 8.20)	0.01 *
Senior high school	1.06 (0.42 to 2.67)	0.89
Family income (per month)		
≥regional minimum wage	2.09 (0.88 to 4.94)	0.09

* Statistically significant.

**Table 3:** Univariate analysis of the factors which were associated with CAS.

Characteristics	CAS (*n* = 41)	No CAS (*n* = 153)	*p*
Subjects (*n* = 194)			
Age (months): mean ± SD	17.0 ± 5.0	16.6 ± 4.7	0.50
Sex			0.05
Male	29 (70.7)	82 (53.6)	
Female	12 (29.3)	71 (46.4)	
Birth weight			0.56
Normal	33 (80.5)	129 (84.3)	
Low birth weight	8 (19.5)	24 (15.7)	
Hemoglobin	9.8 ± 1.0	11.6 ± 1.3	0.00 *
<11 g/dL	(39.0)	115 (75.2)	
≥11 g/dL	64 (61.0)	(24.8)	
Iron-deficiency anemia			0.00 *
Yes	34 (82.9)	35 (22.9)	
No	7 (17.1)	118 (77.1)	
Meal frequency per day			0.68
3 times or more	26 (63.4)	86 (56.2)	
2 times	14 (34.2)	61 (39.9)	
Once	1 (2.4)	6 (3.9)	
Exclusive breastfeeding			0.12
Yes	32 (78.0)	100 (65.4)	
No	9 (22.0)	53 (34.6)	
Current formula milk consumption			0.14
Yes	10 (24.4)	56 (36.6)	
No	31 (75.6)	97 (63.4)	
Weekly meat consumption	1.4 ± 1.3	2.2 ± 2.0	0.06
Weekly consumption of legumes and nuts	3.0 ± 2.7	2.6 ± 2.7	0.36
Weekly egg consumption	3.6 ± 2.8	4.1 ± 2.6	0.344
Weekly milk consumption	2.5 ± 3.2	2.6 ± 3.4	0.94
Mother’s age (years): mean ± SD	30.6 ± 7.4	29.2 ± 6.7	0.27
Gestational age			0.94
Term	36 (87.8)	135 (88.2)	
Preterm	5 (12.2)	18 (11.8)	
Father’s age (years): mean ± SD	34.8 ± 7.9	32.3 ± 7.7	0.17
Mother’s education			0.22
No education	1 (2.4)	1 (0.7)	
Primary school	16 (39.0)	45 (29.4)	
Junior high school	19 (46.3)	64 (41.8)	
Senior high school	4 (9.8)	39 (25.5)	
University	2 (1.9)	3 (3.4)	
Father’s education			0.05
No education	0 (0.0)	1 (0.7)	
Primary school	21 (51.2)	47 (30.7)	
Junior high school	14 (33.5)	51 (33.3)	
Senior high school	5 (12.2)	51 (33.3)	
University	2 (1.9)	2 (2.3)	
Family income (per month)			0.66
≥regional minimum wage	9 (22.0)	29 (19.0)	
<regional minimum wage	32 (78.0)	124 (81.0)	
Weight-for-age			0.00 *
Normal	17 (41.5)	115 (75.2)	
Underweight	11 (26.8)	20 (13.0)	
Severely underweight	13 (31.7)	18 (11.8)	
Weight-for-length			0.07
Normal	27 (65.8)	121 (79.1)	
Overweight	0 (0.0)	1 (0.6)	
Wasted	9 (22.0)	12 (7.8)	
Severely wasted	5 (12.2)	19 (12.4)	

* Statistically significant.

**Table 4:** Multivariate logistic regression analysis of the factors that have a significant association with CAS.

Variables	OR (95 %CI)	*p*
Normal birth weight	1.46 (0.42 to 5.12)	0.55
Weight-for-age		
Underweight	0.93 (0.22 to 3.96)	0.92
Normal	0.48 (0.11 to 2.07)	0.32
Weight-for-length		
Normal	2.59 (0.49 to 13.65)	0.26
Wasted	5.89 (0.95 to 36.45)	0.06
Meal frequency per day		
3 times or more	1.38 (0.52 to 3.63)	0.51
No exclusive breastfeeding	0.69 (0.21 to 2.25)	0.54
Formula milk consumption	0.43 (0.13 to 1.38)	0.16
Preterm gestational age	0.92 (0.19 to 4.39)	0.92
Mother’s education		
Primary school	1.14 (0.14 to 90.26)	0.95
Junior high school	1.38 (0.02 to 106.53)	0.88
Senior high school	0.45 (0.01 to 26.25)	0.70
Father’s education		
Primary school	0.69 (0.01 to 40.27)	0.86
Junior high school	0.37 (0.01 to 20.70)	0.63
Senior high school	0.19 (0.00 to 8.75)	0.40
Family income (per month)		
≥regional minimum wage	4.23 (1.02 to 17.60)	0.05

## Data Availability

The data that support the findings of this study are available from the corresponding author upon reasonable request. Informed consent was obtained from all subjects involved in the study.

## Abbreviations

The following abbreviations are used in this manuscript:

AAP	American Academy of Pediatrics
AOR	Adjusted odds ratio
CAS	Co-occurrence of anemia and stunting
CI	Confidence interval
Hb	Hemoglobin
ID	Iron deficiency
IDA	Iron-deficiency anemia
LBW	Low birth weight
MCH	Mean corpuscular hemoglobin
MCHC	Mean corpuscular hemoglobin concentration
MCV	Mean corpuscular volume
MI	Mentzer index
RBC	Red blood cells
Ret-He	Reticulocyte-hemoglobin
SD	Standard deviation
SLI	Shine and Lal Index
UNICEF	United Nations Children’s Fund
WHO	World Health Organization
